# An illustrated comparison of processing methods for MR phase imaging and QSM: combining array coil signals and phase unwrapping

**DOI:** 10.1002/nbm.3601

**Published:** 2016-09-13

**Authors:** Simon Daniel Robinson, Kristian Bredies, Diana Khabipova, Barbara Dymerska, José P. Marques, Ferdinand Schweser

**Affiliations:** ^1^ High Field Magnetic Resonance Centre, Department of Biomedical Imaging and Image‐Guided TherapyMedical University of ViennaAustria; ^2^ Institute of Mathematics and Scientific ComputingUniversity of GrazAustria; ^3^ Laboratory for Functional and Metabolic ImagingÉcole Polytechnique Fédérale de LausanneSwitzerland; ^4^ Donders Institute for Brain, Cognition and BehaviouRadboud University NijmegenThe Netherlands; ^5^ Buffalo Neuroimaging Analysis Center, Department of NeurologyJacobs School of Medicine and Biomedical Sciences, University at Buffalo, The State University of New YorkNew YorkUSA; ^6^ MRI Clinical and Translational Research CenterJacobs School of Medicine and Biomedical Sciences, University at Buffalo, The State University of New YorkNew YorkUSA

**Keywords:** QSM, phase, array coils, phase combination, phase unwrapping

## Abstract

Phase imaging benefits from strong susceptibility effects at very high field and the high signal‐to‐noise ratio (SNR) afforded by multi‐channel coils. Combining the information from coils is not trivial, however, as the phase that originates in local field effects (the source of interesting contrast) is modified by the inhomogeneous sensitivity of each coil. This has historically been addressed by referencing individual coil sensitivities to that of a volume coil, but alternative approaches are required for ultra‐high field systems in which no such coil is available. An additional challenge in phase imaging is that the phase that develops up to the echo time is “wrapped” into a range of 2*π* radians. Phase wraps need to be removed in order to reveal the underlying phase distribution of interest.

Beginning with a coil combination using a homogeneous reference volume coil – the Roemer approach – which can be applied at 3 T and lower field strengths, we review alternative methods for combining single‐echo and multi‐echo phase images where no such reference coil is available. These are applied to high‐resolution data acquired at 7 T and their effectiveness assessed via an index of agreement between phase values over channels and the contrast‐to‐noise ratio in combined images. The virtual receiver coil and COMPOSER approaches were both found to be computationally efficient and effective.

The main features of spatial and temporal phase unwrapping methods are reviewed, placing particular emphasis on recent developments in temporal phase unwrapping and Laplacian approaches. The features and performance of these are illustrated in application to simulated and high‐resolution *in vivo* data. Temporal unwrapping was the fastest of the methods tested and the Laplacian the most robust in images with low SNR. © 2016 The Authors. *NMR in Biomedicine* published by John Wiley & Sons Ltd.

**Abbreviations used**
CNR: contrast‐to‐noise ratio
*COMPOSER*: *combining phased array data using offsets from a short echo‐time reference*
*DCT*: *discrete cosine transform*
*EPT*: *electric property tomography*
*GM*: *gray matter*
*HiP*: *Hermitian inner product*
*MAGPI*: *maximum ambiguity distance for phase imaging*
*MCPC‐3D‐II*: *multi‐channel phase combination using 3D phase offsets derived from a separate dual echo scan*
*PCG*: *preconditioned conjugate gradient*
*QSM*: *quantitative susceptibility map/mapping*
*ROI*: *region of interest*
*SENSE*: *sensitivity encoding for fast MRI*
*SNR*: *signal‐to‐noise ratio*
*SPM*: *scalar phase matching*
*SVD*: *singular value decomposition*
*SWI*: *susceptibility‐weighted imaging/image*
*UHF*: *ultra‐high field*
*UMPIRE*: *unwrapping multi‐echo phase images with irregular echo spacings*
*VBC*: *virtual body coil*
*VRC*: *virtual reference coil*
*VRI*: *virtual reference image*
*WM*: *white matter*

## Introduction

**Figure 1:**
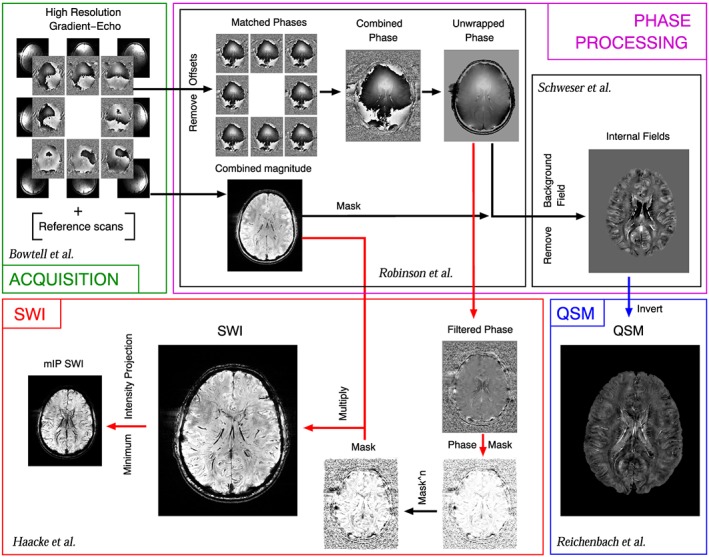
The main steps in the analysis pipeline for SWI and QSM. References relate to this and other reviews in this special issue.

Although phase information is frequently disregarded in MRI, every MR measurement yields phase data as the inherent counterpart to the magnitude information arising from the Fourier transform of the complex‐valued resonance signal. The phase of *T*_2_*‐weighted gradient echo scans contains a particular richness of anatomical and biophysical information, as has been outlined in other contributions to this special edition. This review focuses on preprocessing steps required to generate combined, artifact‐free phase images from multi‐channel coils. Its counterpart, by Schweser *et al.* 1, considers approaches to the removal of the background field. These steps precede the generation of quantitative susceptibility maps (QSMs) 2, 3 and susceptibility‐weighted images (SWIs) 4, 5 and are labeled “phase processing” in the overview of a typical pipeline for the generation of QSMs and SWIs shown in Figure 1.

The phase differs from the magnitude in some important aspects, which means that it requires dedicated methods to allow the information it contains to be faithfully identified. Outside the area of the region of the image occupied by the object, for instance, noise voxels have the same range of values as voxels in the object itself, meaning that the object is inherently more difficult to discern. The phase image is also subject to *wraps* when the phase, which has evolved from excitation to the echo time, falls outside a range of 2*π* rad. The phase measured with each coil in an RF array is also subject to the sensitivity of that coil, making it problematic to combine signals from a number of coils and preserve phase information arising from susceptibilities in the object.

Many of the topics covered here have been addressed in a number of other recent reviews 3, 6, 7. Our aim in this article and the related review by Schweser *et al.* in this special issue is to complement those by providing a practical, illustrated guide to phase processing. By applying the most relevant and effective methods for phase combination, unwrapping and background field removal to simulated and ultra‐high‐field (UHF) *in vivo* data, we hope to afford the reader with clear guidelines as to the most suitable phase processing choices for a wide range of QSM and other phase contrast applications.

## Combining Multi‐Channel Phase Images

An assembly of RF coils, each with its own amplifier and receiver channel and designed for simultaneous reception of signal, is called a ‘phased array’ 8. Phased array coils provide higher signal‐to‐noise ratio (SNR) than volume coils 8, allow the acquisition to be accelerated using parallel imaging 9, 10, 11, 12 and enable control over patterns of transmit RF (*B*_1_^+^) via parallel transmit excitation 13, 14. These features are particularly beneficial at ultra‐high static magnetic field (7 T and above) due to inhomogeneous *B*_0_ and *B*_1_ fields.

Complex signals from each coil in a phased array can be combined at a number of stages in the QSM processing. At one extreme, signal can be acquired and processed, from *k*‐space signal to QSM, entirely separately for each channel, and an average QSM over all channels calculated 15. This circumvents the problem of combining raw coil raw phases, which have disparate values due to inhomogeneity in the coil sensitivities, but is computationally demanding, particularly as the number of coils in arrays increases. At the other extreme, coil signals can be combined at the beginning of the receive chain, so that only the combined signals are digitized and recorded 16. This reduces data storage and processing requirements but can lead to destructive interference between the complex coil signals. The approaches in the following sections all combine phase information from signals which are acquired and reconstructed into separate complex images for each channel. This is the earliest stage in the processing pipeline at which it is possible to apply spatially varying (i.e. non‐constant) corrections for the sensitivity of each coil.

### The phase measured with a single RF coil

The phase measured with a single RF coil comprises both an echo‐time‐dependent contribution and a time‐independent contribution. The time‐dependent contribution arises from local changes to the static magnetic field, ∆*B*_0_, which originate, at least in part, from tissues with different magnetic susceptibilities. The phase that accrues due to these effects in a measurement with *K* echoes, at *TE*_*k*_, is

(1) $$\varphi \left(\vec{r},{TE}_{k}\right)=\varphi^{0}\left(\vec{r}\right)+2\pi {\gamma {TE}_{k}∆B}_{0}\left(\vec{r}\right),$$

where $\varphi^{0}\left(\vec{r}\right)$ is the echo‐time‐independent *phase offset*, sometimes referred to as the *transceive phase*, *γ* the gyromagnetic ratio and $\vec{r}$ the spatial location vector. Equation (1) neglects measurement noise and second‐order, non‐linear phase evolution, which manifests if tissues contain multiple water compartments with different resonant frequencies 17, 18, 19. The term $\varphi^{0}\left(\vec{r}\right)$ is generally taken to include all contributions to the phase, including coil sensitivity, which would be measured at a nominal echo time of *TE* = 0 ms.

The phase that has developed by time *TE*_*k*_, $\varphi \left(\vec{r},TE_{k}\right)$, is ‘wrapped’ into the inherent encoding range of 2*π*. The limits of the range are given by (*φ*_L_, *φ*_L_ + 2*π*], where *φ*_L_, the lower limit, can be chosen at will but is usually ascribed the value –*π* or 0. At a given position and *TE*_*k*_, the measured, or wrapped, phase *Φ* is given by

(2) $$\Phi =\text{φmod}2\pi +\varphi_{L}=\varphi -2\pi \left\lfloor \left.\frac{\varphi }{2\pi }\right\rfloor \right.+\varphi_{L}$$

where mod denotes the modulo operation and the straight brackets indicate rounding down. Wraps are phase isocontours, which should form closed loops within the object or begin and terminate at the object boundary.

**Figure 2:**
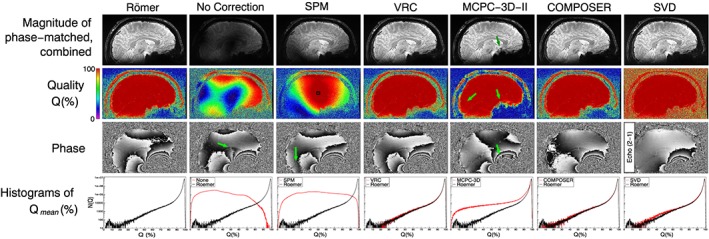
Comparison of phase combination outcomes for the methods under consideration. The absolute value of the phase‐matched complex sum for Echo 2 ‘Magnitude of phase‐matched, combined’ (as in Equation (5) in Reference 55) is shown for each method (other than for the SVD method, where the difference image is shown for Echo 2 − Echo 1), as well as the phase matching quality index (*Q*), and the combined phase image (all sagittal). The histogram of the mean *Q* over all echoes and all voxels in the brain is shown for each method (red line), with the *Q*_mean_ results of the Roemer shown for reference (black line) (note logarithmic scale on the vertical axis).

Wraps which terminate within the object are often referred to as ‘open‐ended fringelines’ (see, e.g., the phase image in Figure 2, with ‘No Correction’ at the arrow position). Open‐ended fringelines are characterized by the presence of a ‘residue’, or loop of 2 × 2 voxels at the object‐end termination of the fringeline for which the sum of the wrapped phase differences is not zero. Open‐ended fringelines cannot be unwrapped by adding an integer value of 2*π* to a circumscribed region, as the residue poses an unwrapping paradox analogous to the Penrose stairs (made famous by Escher's drawings) 20. Residues occur where the phase evolution between neighboring voxels is greater than *π* (which is more likely to occur at low resolution and where there is a large Δ*B*_0_ *T*_E_ product) or where noise dominates 21, 22. In this latter case, the signal magnitude is close to zero. In a single coil, this can occur because the sensitivity of the coil is low, either due to the distance from the object, because the transverse component of the receive field is zero at the given point or due to coupling with other coils. In combined phase images, the combination of complex data from many coils may sum to zero magnitude (and arbitrary phase) due to interference between the signals. The reader is referred to References 21 and 22 for more detailed explanations of this phenomenon.

The wraps that affect the measured phase frustrate the determination of ${\text{∆}B}_{0}\left(\vec{r}\right)\;$and also $\varphi^{0}\left(\vec{r}\right)\;$in some contexts. Approaches to recovering the underlying phase by *unwrapping* are described in the next section.

### The phase measured in each element of a RF array coil

In the context of processing phased array data, it is important to recognize that the phase component that accrues from ∆*B*_0_ is common to the signal in all coils in an array, whereas the phase offset, ${\varphi_{j}}^{0}\left(\vec{r}\right)$, is different for each coil in the array. That is, for the set of *J* array coils, Equation (1) has to be rewritten as

(3) $$\varphi_{j}\left(\vec{r},{TE}_{k}\right)=\varphi_{j}^{0}\left(\vec{r}\right)+2\pi \gamma {TE}_{k}{∆B}_{0}\left(\vec{r}\right).$$

The *phase offset*, $\varphi^{0}\left(\vec{r}\right)$, comprises contributions that are common to all coil signals and contributions that are different for each coil in the array. Contributions that are common to all coils include *B*_1_^+^ phase, eddy current effects, Maxwell terms and a phase gradient in the readout direction caused by mistiming between the gradient and the acquisition 23. In contrast to these effects, the signal measured by each coil is modified by a complex‐valued function that reflects the object distance from the coil and the RF wavelength 24: the receive sensitivity or *B*_1_^−^. This, and a constant term reflecting the length of the receiver chain, are different for each coil in the array, and need particular attention as they lead to destructive interference in a complex sum of coil signals. The spatial variation of the receive sensitivity of each coil is determined by the interaction with the signals from other coils (coupling) and the wavelength of RF radiation in tissue, which at high field is of the order of size of the brain (circa 30 cm at 3 T and 12 cm at 7 T 25). Given the conductivity and permittivity of the object, this could, in principle, be modeled for each subject on the basis of the position relative to the coils during measurement adjustment, and used to address the problem of phase combination, although in practice calculations with large arrays are complex and demanding 26. The sign of eddy current and readout gradient phase is dependent on whether signal is acquired during positive or negative readout gradient lobes, leading some authors to specify this contribution in a dedicated term *φ*_*l*(*k*)_, where for bipolar acquisitions *l*(*k*) = 1 when *k* is odd and *l*(*k*) = 2 when *k* is even 27. Equation (3) and further descriptions here apply to monopolar acquisitions and all even or all odd echoes in a bipolar acquisition.

In the context of QSM, $\varphi^{0}\left(\vec{r}\right)$ is considered to contain no interesting information and is treated as a nuisance effect. In fact, the phase distribution of the *B*_1_ field allows electrical properties such as conductivity and permittivity to be quantified, an approach known as electric property tomography (EPT) 28. While EPT is beyond the scope of this review, readers interested in EPT may find the methods, which allow $\varphi^{0}\left(\vec{r}\right)$ to be determined explicitly, to be of interest.

It is clear from Equation (3) that subtracting the phase offset, $\varphi_{j}\left(\vec{r},TE_{k}\right)-\varphi_{j}^{0}\left(\vec{r}\right)$, eliminates the coil‐dependent term. We will refer to this process, which is the basis of combining phase information from a number of coils, as ‘matching’ the coil phases. The remaining, susceptibility‐related phase, $\vartheta_{j}\left(\vec{r},TE_{k}\right)$, is the same for all coils.

Equation (3) can be solved uniquely for multi‐echo measurements (see later in this section), but is underdetermined for single‐echo acquisitions (*K* = 1). Solutions for this case require additional information from a scan with a reference coil or involve assumptions about the smoothness of $\varphi_{j}^{0}\left(\vec{r}\right)$. These approaches are also described later in this section.

### Common operations on phase

The methods in the following sections involve some operations on the phase which it is worthwhile to define here explicitly, to provide readers new to the field with a starting point for implementing phase combination methods.

In phase imaging one often needs to calculate the difference between two wrapped phase measurements of *φ*, *Φ*_*n*_ and *Φ*_*m*_. Subtracting the two, *Φ*_*m*_ − *Φ*_*n*_, propagates wraps present in both *Φ*_*n*_ and *Φ*_*m*_ into the difference. The *complex difference*, *Φ*_*m* − *n*_, on the other hand, is only wrapped where the difference between the (unknown) unwrapped phases, *φ*_*m*_ − *φ*_*n*_, is outside the range (*φ*_L_ − *π*, *φ*_L_ + *π*]. The complex difference can be formulated as

- the angle of the difference between the two wrapped phase values in complex exponential form,

(4) $$\Phi_{m-n}=∠\exp\left(i\left(\Phi_{m}-\Phi_{n}-\varphi_{L}\right)\right)+\varphi_{L}$$

where ∠ denotes the four‐quadrant tangent inverse (which is usually called atan2 in computer languages), or

a simple subtraction of the second angle from the first, with 0, 2*π* or −2*π* being added to the difference, depending on the relative sizes of the two angles:

(5) Φm−n=Φm−Φn,ifφL−π<Φm−Φn<φL+πΦm−Φn−2π,ifΦm−Φn>φL+πΦm−Φn+2π,ifΦm−Φn<φL−π.

The second expression (Equation (5)) is particularly useful to avoid calculations involving complex numbers, e.g. if the available computer memory is limited.

The maximum and minimum effective frequencies in the rotating frame, Δ*f*_MR , max_ and Δ*f*_MR , min_, which are not wrapped in the difference image *Φ*_*m* − *n*_, are given by (Δ*f*_MR , max_ − Δ*f*_MR , min_) × (*TE*_*m*_ − *TE*_*n*_) < 1. The significance of *φ*_L_ in Equations. (2)–(4), which is usually omitted from the literature, is that it may be chosen judiciously to extend the region of the image over which *Φ*_*m* − *n*_ is wrap free. This is pertinent in calculating the difference between phase images acquired at two echo times in the brain, for instance, where (despite shimming) the ∆*B*_0_ values superior to *Z* ≅  − 20 in MNI space 29 are not distributed equally about zero, but skewed to either positive or negative values, depending on the vendor's convention 30. For example, effective frequencies in the rotating frame of −50 Hz to +50 Hz lead to a complex phase difference without wraps for 10 ms if *φ*_L_ = 0. Choosing *φ*_L_ = *π*/2 would change this range to −25 Hz to +75 Hz, which may be more appropriate for the range of frequencies encountered. Practically, this may allow a wrap‐free phase difference image to be generated for a particular region and echo time difference if typical values of ∆*B*_0_ are known.

Another common operation in phase processing is smoothing, either for noise reduction or as a step in high‐pass filtering 31. Smoothing of wrapped phase needs to be performed in the complex representation to avoid blurring wraps: i.e., the real and imaginary components should be smoothed independently. The smoothed phase *Φ*_s_ can be expressed as *Φ*_s_ =  ∠ (*S*(*M* sin (*Φ*)) + i*S*(*M* cos (*Φ*))), where *M* is the magnitude and *S* is an (unspecified) smoothing function.

### Phase combination solutions where there is a reference coil: the Roemer/SENSE approach

If the MR system has a coil with homogeneous receive sensitivity, such as a body volume coil, the sensitivities of each element of the phased array can be measured with respect to the sensitivity of this 8. A similar approach is used with sensitivity encoding for fast MRI (SENSE) reconstruction, which likewise generates phase and magnitude images with close‐to‐optimum SNR 9, 32. Rather than matching the phases of the coil signals using the phase offset, $\varphi_{j}^{0}\left(\vec{r}\right)$, the complex signals in a voxel are generally combined to a complex image value *P*, given by *P* = *λ****p***^T^***R***^−1^***b***, where ***p*** is a vector of complex‐valued signal values of all coils in a certain voxel, the vector ***b*** contains the complex‐valued coil sensitivities in the voxel, *λ* is a scaling factor and ***R*** is the noise correlation matrix 8. For a number of coils *J*, ***R*** is a *J* × *J* matrix in which the diagonal elements are the noise levels for each coil and the off‐diagonal elements are the correlations between coils *x* and *y* (where *x* and *y* run from 1 to *J*) 33. ***R*** can be determined from a prescan with no RF excitation. The scaling factor *λ* is often set to unity, but can be used to correct for inhomogeneities in the sensitivity of the reference volume coil.

While this approach is useful at clinical field strengths, UHF MR scanners usually do not have a body volume coil with which to perform the reference measurement needed for the Roemer approach. Some transmit coils are engineered to be able to receive signal for this purpose (i.e. to operate as transceive coils), but these generally do not have very homogeneous sensitivity at UHF. This does not detract from the quality of phase matching, but introduces the inhomogeneous *B*_1_^−^ phase of the transceiver coil, in addition to the already present *B*_1_^+^ phase, into the combined image. This can affect the QSM, if not removed 34, 35. More problematic is the fact that for many coils, particularly the forthcoming generation of parallel transmit coils, it may be costly or impractical to engineer the transmit array to receive signal due to the need to include transmit–receive switches and preamplifiers. Receiver channels also need to be allocated to these coils, which may limit the number of elements available to the receive‐only array. The additional electronics may also disrupt the other elements in the coil, particularly if the array includes *B*_0_ shimming coils 36. These considerations, and the sensitivity of this method to motion between the acquisition of sensitivity maps and the scan of interest, provide the motivation for the following phase combination methods, none of which requires a volume coil reference measurement.

### Reference‐coil‐free solutions for single‐echo acquisitions

#### Frequency filtering (homodyne filtering and unwrap‐and‐filter)

Spatial frequency filtering approaches to phase combination were developed for susceptibility‐weighted imaging (SWI). The increasing use of UHF, multi‐channel coils and methodological developments in the field have allowed a move from SWI to phase imaging 37, phase value quantification 38, 39, 40 and quantitative susceptibility mapping (QSM) 2, 41, 42. This has been accompanied by a shift in focus from small veins to iron‐containing gray matter (GM) structures, including those of several millimeters in size, such as the putamen and pallidum 43. The modified contrast generated by frequency filtering for SWI confounds QSM, as QSM interprets phase quantitatively in the ill‐posed inverse problem. Frequency filtering methods are included in this review to cover the historical development of phase combination and for completeness.

*In vivo* phase images comprise a wide range of spatial frequencies. The macroscopic changes to the local magnetic field arising from the interfaces between tissues with large susceptibility differences such as bone, GM, cerebro‐spinal fluid and air, particularly in inferior and frontal regions, are dominated by low spatial frequencies, as are phase offsets. Venous vessels – the original focus of interest in venography 4, 5 and SWI 44 – give rise to high spatial frequencies. This offered the attractive prospect of being able to suppress both phase offsets and macroscopic susceptibility effects by high‐pass filtering the data.

The simplest means to achieve this is to divide the complex image data for each channel by a smoothed version of the same data 45. This *homodyne filtering* not only removes phase offsets but also reduces the effective phase range, removing wraps in the process. While this is quite effective in superior brain regions at low and intermediate fields, it often fails in regions with very strong field gradients, such as regions close to the sinus cavities, where the high‐pass filtering is not sufficient to reduce the filtered phase to the range (0, 2*π*]. The process needs to remove all wraps, as any residual wraps take the form of open‐ended fringelines, which cannot be subsequently unwrapped by conventional means (see the next section). This constraint leads to the need to apply relatively strong filtering at high field strength, where ∆*B*_0_*TE* is generally higher, leading to heavily modified contrast and often still leaving residual wraps in frontal and ventral regions, making the resulting phase images unusable for QSM. As unsatisfactory as this solution is at very high field, it is still frequently used in commercial MR systems. An improvement to the method is offered by the *unwrap‐and‐filter approach*, introduced for single‐channel data at 3 T by Rauscher *et al.* 31 and later applied to combining array coil UHF data by Koopmans *et al.* 46. Here, phase images from each channel are spatially unwrapped then high‐pass filtered before being combined using a magnitude‐weighted mean. The removal of wraps allows greatly reduced filtering to be used: only that needed to remove phase offsets. The need to unwrap phase images is a drawback, however, as this process is computationally demanding and prone to errors in the presence of phase noise, which is particularly problematic with small surface coils with inhomogeneous sensitivity profiles. Applying Laplacian unwrapping rather than path‐following spatial unwrapping (see next section) could prove advantageous 15, but with the unwrap‐and‐filter method having been superseded by others described in this section this seems not to have been widely pursued.

#### Scalar phase matching

A simple approach to estimating the channel‐dependent phase offsets, $\varphi_{j}^{0}\left(\vec{r}\right)$, to match the phase images, is to approximate them to channel‐dependent constants 47, 48. This has been variously called scalar phase matching (SPM), multi‐channel phase combination using constant phase offsets (MCPC‐C) or the Hammond method.

SPM estimates the offset value for each channel from the mean phase value in a matching region of interest, ROI_M_. The estimate $\Theta_{j}\left(\vec{r},{TE}_{k}\right),$ of susceptibility‐related phase in each channel, $\vartheta_{j}\left(\vec{r},{TE}_{k}\right),$ is

(6) $${\Theta_{j}\left(\vec{r},{TE}_{k}\right)=\varphi_{j}\left(\vec{r},{TE}_{k}\right)-\overset{-}{\varphi }}_{j}\left({\mathrm{ROI}}_{M}\right),$$

where ${\overset{-}{\varphi }}_{j}\left({\mathrm{ROI}}_{M}\right)$ is the mean phase over ROI_M_. The choice of ROI_M_ is problematic. There must be sufficient signal in all coils in ROI_M_ to estimate $\varphi_{j}^{0}$ at that position. This requires there to be a point of mutual detection. ROI_M_ should be close to the center of the object to minimize the error in estimating the phase offsets, $\varphi_{j}^{0}\left(\vec{r}\right)-{\overset{-}{\varphi }}_{j}\left({\mathrm{ROI}}_{M}\right)$, and ROI_M_ should not contain wraps 49.

Hammond *et al.* chose ROI_M_ to be at the center of the image 48. Schäfer and Turner noted the problems that arise when there is no signal at the center of the image 50 and suggested that ROI_M_ be centered on the voxel in which the product of the single‐channel magnitude images over coils is a maximum 50. This ensures that there is signal in all coils at this position, but ROI_M_ may be far from the object center.

The fundamental problem with SPM, however, is that the approximation that the phase offsets, $\varphi_{j}^{0}\left(\vec{r}\right)$, are constant over the image is not well adhered to at UHF. The error in this approximation, $\varphi_{j}^{0}\left(\vec{r}\right)-{\overset{-}{\varphi }}_{j}\left({\mathrm{ROI}}_{M}\right)$, increases with spatial variation in $\varphi_{j}^{0}\left(\vec{r}\right)$, i.e. shorter wavelength at higher field, and distance from ROI_M_. The regional loss of SNR in combined phase images may only be apparent with a larger number of coils (the original work was with an eight‐channel coil 48), and when a quantitative index of phase matching quality is employed (such as *Q*, introduced later in this section). SPM can break down completely, though, and lead to an open‐ended fringeline at a position that depends on the RF wavelength in tissue (i.e. the field strength), the object size and the number and arrangement of coils. Our experience is that this happens circa 5 cm inferior to ROI_M_ with a 32‐channel coil at 7 T. As will be seen in the next section, wherever SPM generates a combined image without open‐ended fringelines, a virtual coil method can improve on this.

#### Virtual coil methods

In the absence of a body coil, a virtual reference image (VRI) can be generated from the array coil data itself. In virtual coil methods, the phase of each coil in the array is referenced to that of the VRI, similar to the body‐coil image in the Roemer technique.

The virtual body coil (VBC) 16, 51 and virtual reference coil (VRC) 52 methods differ in how the virtual image is generated. In the VBC approach, the VRI is the dominant singular vector in a singular value decomposition (SVD) of the array coil data. Originally conceived as a means of reducing the volume of data needing to be reconstructed 16, it was extended to use the VBC as a reference for phase combination 51. The robustness of the VBC approach with different coil designs and field strengths remains to be investigated. In the VRC method, the VRI is generated using SPM.

In both the VBC and VRC approaches, the phase differences between each channel and the VRI are complex smoothed (see earlier in this section) and subtracted from the respective channels to phase match the signals. It might be supposed that any choice of VRI would yield a perfectly matched result. This is not the case, and interesting insights into the phase combination problem are provided by consideration of the choice of the VRI. If the VRI is taken to be zero in all voxels, for instance, the difference between the signal from each channel and that of the VRI is simply the signal from each channel itself, and the VRC approach is equivalent to homodyne filtering – the limitations of which have been discussed above. The sophistication in the VRC approach lies in the fact that the VRI, despite having low SNR away from the center (see earlier in this section), contains all the phase *contrast* common to all channels, so the difference between each channel and the VRI is small (generally not wrapped) and smooth.

The practical appeal of the VRC approach is that neither a reference coil measurement nor other reference scans are needed, and that deficiencies in the phase matching in the SPM method are remedied in the second step (phase matching to the VRI), as long as the phase in the VRI is well defined. The VRC fails, however, when there are open‐ended fringelines in the VRI. That is, it breaks down wherever the SPM method itself breaks down. As such, while it yields phase images with higher SNR than SPM – particularly at a distance from ROI_M_ – it is not more robust than SPM.

More sophisticated solutions for generating a VRI are required for large objects at 7 T, such as the abdomen, and for neuroimaging at higher field strengths. One possibility would be to modify the SPM approach so that, rather than using a set of phase correction constants that yield perfect phase matching only at ROI_M_, a set of *J* complex scalar weights could be determined that would yield homogeneous (but imperfect) phase matching throughout the object. This is the receive equivalent of RF shimming in parallel transmission 13, 53.

It should be noted that, while phase matching can be excellent with virtual coil methods, the phase corrections applied to each channel are not equal to $\varphi_{j}^{0}\left(\vec{r}\right)$. The fact that the VRI is inhomogeneous means that arbitrary phase fluctuations are introduced into both sides of Equation (3), and the combined phase is subject to the same phase variation, which generally varies slowly in space. In conclusion, the virtual coil approach can always be applied when SPM can be applied, but provides better phase matching. As such, SPM should only be used to generate a VRI (subject to the provisos above) and not the final combined phase image.

#### Multi‐channel phase combination using 3D phase offsets derived from a (separate) dual echo scan (MCPC‐3D‐II)

In the absence of wraps, the phases of two images acquired at *TE*_1_ and *TE*_2_ can be used to calculate the phase offsets of each channel *j* (see Equation (3) and Reference 54):

(7) $$\varphi_{j}^{0}\left(\vec{r}\right)=\frac{\varphi_{j}\left(\vec{r},{TE}_{2}\right)TE_{1}-\varphi_{j}\left(\vec{r},{TE}_{1}\right)TE_{2}}{{TE}_{1}-{TE}_{2}}.$$

Although this approach uses temporal evolution of the phase, we include it under combination approaches for single‐echo phase imaging because, in the MCPC‐3D‐II variant of this method, $\varphi^{0}\left(\vec{r}\right)\;$are determined from a separate (low‐resolution) dual‐echo acquisition but applied to a single‐echo scan. Combined phase images with this method are free of $\varphi^{0}\left(\vec{r}\right)$.

The main drawbacks with this approach are vulnerability to motion between acquisitions, the need to coregister (or upscale) the low‐resolution maps of $\varphi^{0}\left(\vec{r}\right)\;$with the data to be combined and the need to spatially unwrap phase images. This final consideration makes it problematic to implement on the scanner console and renders it sensitive to errors in low‐SNR regions.

#### Combining phased array data using offsets from a short echo‐time reference (COMPOSER)

The simple observation underlying coil combination with this method is that the term $2\pi \gamma TE_{k}{\text{∆}B}_{0}\left(\vec{r}\right)$ in Equation (3) tends to zero as *T* *E*_*k*_ tends to zero, at which point $\varphi_{j}\left(\vec{r},{TE}_{k}\right)$ approximates to $\varphi_{j}^{0}\left(\vec{r}\right)$. Whereas MCPC‐3D‐II extrapolates the temporal evolution of the phase to *TE* = 0 to identify phase offsets, COMPOSER measures an approximation to them directly 55. It is practical to acquire the short‐echo‐time reference phase data, $\varphi_{\mathrm{ref},j}\left(\vec{r},TE{,}_{\text{SHORT}}\right)$, in a fast, low‐resolution scan with a short‐*TE* sequence such as vTE 56, UTE 57, PETRA 58 or a conventional GE scan with short *TE* (~1–2 ms; using a short excitation pulse, high bandwidth and asymmetric echo), and apply this to high‐resolution single‐echo or multi‐echo data. The steps in COMPOSER are coregistration or upscaling of $\varphi_{\mathrm{ref},j}\left(\vec{r},TE{,}_{\mathrm{SER}}\right)\;$to the high‐resolution scan, and subtraction from the high‐resolution data of interest.

(8) $$\Theta_{j}\left(\vec{r},TE_{k}\right)=\varphi_{j}\left(\vec{r},TE_{k}\right)-\varphi_{\mathrm{ref},j}\left(\vec{r},TE{,}_{\mathrm{SER}}\right).$$

COMPOSER requires reference data, and, like MCPC‐3D, needs this to be coregistered to the scans of interest. Without the need to unwrap phase data it has significant advantages over MCPC‐3D in matching quality, robustness and computational load, and should supplant MCPC‐3D in all applications.

### Reference‐coil‐free solutions for multi‐echo acquisitions

When phase data is acquired at multiple echo times, the temporal evolution of the signal over the echoes can be used to retrieve the phase offset. Methods found in literature to compute phase images can be classified into two groups: those that reconstruct *phase difference images* and those that reconstruct *phase images* for each echo.

#### Phase difference methods

One approach to eliminating the channel‐dependent phase offsets, $\varphi_{j}^{0}\left(\vec{r}\right)$, is to calculate a phase difference for each coil using the phase from one or more pairs of echoes acquired at two different echo times *TE*_*n*_ and *TE*_*m*_ 59, 60, 61.

Complex signals $I_{j}\left(\vec{r},TE_{k}\right)=m_{j}\left(\vec{r},TE_{k}\right)e^{-i\vartheta \left(\vec{r},TE_{k}\right)}$ can be combined over channels in the Hermitian inner product (HiP) to generate estimations of the phase evolution between echoes *n* and *m*, $\Theta^{d}\left(\vec{r},n,m\right):$

(9) $$\Theta^{d}\left(\vec{r},n,m\right)=∠\left[\sum_{j}\kappa_{j}^{d}\;I_{j}\left(\vec{r},{\mathrm{TE}}_{n}\right)I_{j}^{+}\left(\vec{r},{\mathrm{TE}}_{m}\right)\right]=∠\left[\sum_{j}\kappa_{j}^{d}\;\left|m_{j}\left(\vec{r},{\mathrm{TE}}_{n}\right)\right|\left|m_{j}\left(\vec{r},{\mathrm{TE}}_{m}\right)\right|e^{-i\left(\varphi \left(\vec{r},{\mathrm{TE}}_{n}\right)-\varphi \left(\vec{r},{\mathrm{TE}}_{m}\right)\right)}\right],$$

where $\kappa_{j}^{d}$ is a weight that accounts for varying noise in different coil channels 61. This method can be performed without the requirement of unwrapping the phase image for each channel, but the SNR is reduced because the weights are retrieved from the images themselves and contain noise. Smoothing the weights decreases the noise as long as the coil sensitivities are not corrupted 62.

When data is acquired with multiple echoes, the SVD method (see Appendix A1) can be applied to the data from all echoes to calculate the coil sensitivities, resulting in an optimum SNR for both the magnitude and phase 63. This approach has been shown to be effective for spectroscopy data 64. The pixel‐by‐pixel SVD factorization of the channel–echo time matrix combines the data from the different coils. Hereby, the first singular value is the maximal coherently constructed signal from all channels and echoes and the eigenvectors contain the coil sensitivity estimations as well as the complex signal, *S*, of the acquired echoes. The phase of the complex data has an arbitrary offset due to the pixel‐by‐pixel nature of the method, but not the phase differences between the first echo and each subsequent echo.

Combined field maps are calculated using the unwrapped phase difference between each different echo acquired at *TE*_*k*_ and the first echo *TE*_1_. The final field map is obtained using weighting factors

(10) $$W^{d}\left(\vec{r},k\right)=\frac{{M^{d}\left(\vec{r},k\right)}^{2}}{{M^{d}\left(\vec{r},k\right)}^{2}+{M^{d}\left(\vec{r},1\right)}^{2}}$$

as in Reference 65, where *M*^*d*^ is the absolute value of the signal evolution between Echo 1 and echo *k*. This results in the field map:

(11) $$∆B\left(\vec{r}\right)=\left(\frac{1}{2\pi \gamma }\right)\frac{\sum_{k=2}^{K}\;\Theta^{d}\left(\vec{r},k\right)\left({TE}_{k}-{TE}_{1}\right)\cdot W^{d}\left(\vec{r},k\right)}{\sum_{k=2}^{K}{\left({TE}_{k}-{TE}_{1}\right)}^{2}\;W^{d}\left(\vec{r},k\right)}$$

(see Appendix A2 for a derivation). Note that the weighting factors for *phase difference images* differ from those for *phase images* (see later in this section).

#### Phase imaging methods

Instead of cancelling the phase offsets, $\varphi_{j}^{0}\left(\vec{r}\right)$, by using the phase difference, MCPD‐3D‐I calculates them for each channel using a multi‐echo scan 54, as described earlier in this section. This has the advantage over the HiP approach that $\varphi_{j}^{0}\left(\vec{r}\right)$ can be smoothed before being subtracted from the phase at each echo time, leading to a higher SNR result. The computational complexity may be mitigated either by using a low‐resolution multi‐echo scan to calculate the phase offsets (applying MCPC‐3D‐II, described earlier in this section, to the multi‐echo data) or by downsampling the high‐resolution data for the phase offset calculation step, but it is nonetheless subject to the shortcomings of phase unwrapping.

In the MAGPI approach (maximum ambiguity distance for phase imaging) 66, the measured phase from Equation (3) is extended to

(12) $$\varphi_{j}\left(\vec{r},{TE}_{k}\right)=\varphi_{j}^{0}\left(\vec{r}\right)+2\pi \gamma TE_{k}{∆B}_{0}\left(\vec{r}\right)+\varphi_{\mathrm{jk}}^{\text{noise}}+\varphi_{\mathrm{jk}}^{\text{wrap}}$$

where *φ*_*jk*_^noise^ denotes the additive noise for each coil *j* and echo time *TE*_*k*_ and *φ*_*jk*_^wrap^ denotes 2*π* phase wraps.

This method uses the likelihood function, resulting in an increase of the SNR in the case of a three‐echo measurement. The reconstruction of the corrected phase image is performed in three steps. In the first step, an estimation of the most likely tissue‐based frequency, which describes the phase difference between echoes, is calculated and removed from the original data. The angle difference is assumed to be $\Theta^{d}\left(r,n,m\right)=2\pi {\text{∆}B}_{0}\left(\vec{r}\right)\left({TE}_{2}-{TE}_{1}\right)+\left(\varphi_{j}^{2}-\varphi_{j}^{1}\right)+2\mathrm{\pi R}$, where the phase wrapping is assigned the integer *R*, which forces the angle difference to be in the range ( − *π* , *π*].

The remaining data is associated with random noise, *φ*_*j*_^n^, as well as the phase offset, $\varphi_{j}^{0}\left(\vec{r}\right)$, which are separated in the second step. Finally, the most likely tissue frequency that explains the three echoes is calculated. This method estimates the underlying phase without phase unwrapping or denoising and outperforms previous methods for measurements with low SNR, with the drawback of being computationally intensive.

In summary, the phase difference method using the HiP approach can be used to combine the phase images from multiple channels. This can be performed very quickly without the need for phase unwrapping, although the SNR of the combined phase image is reduced by the voxel‐by‐voxel subtraction. Using the 3D correction of the coil sensitivities improves the reconstructed phase image at the cost of computationally intensive phase unwrapping for each channel and the two echoes. The whole dataset is used for the calculation of the complex signal evaluation for the SVD method. Both phase unwrapping and phase offset are incorporated in the maximal likelihood (MAGPI) calculation, which performs better at low SNR.

### Combining matched phase signals over channels and echoes

In the previous sections we have looked at ways to estimate or approximate phase offsets. The individual phase images can be phase matched by subtraction of the phase offset contribution: $\Theta_{j}\left(\vec{r},TE_{k}\right)=\varphi_{j}\left(\vec{r},TE_{k}\right)-\varphi_{j}^{0}\left(\vec{r},TE_{k}\right)$. The matched phases from each coil must then be combined over channels. Optimal SNR is achieved by including both coil sensitivities and consideration of noise correlation between signals from different channels, as described in the *spatially matched filter* approach 8, 67. In combining phases, many authors omit the effects of noise correlation and simply sum the phase values in complex exponential form, with weights $\kappa_{j}\left(\vec{r},TE_{k}\right)$:

(13) $$\Theta \left(\vec{r},{TE}_{k}\right)=∠\left[\sum_{j}\kappa_{j}\left(\vec{r},{TE}_{k}\right)e^{-i\Theta_{j}\left(\vec{r},{TE}_{k}\right)}\right].$$

The use of magnitude squared values for $\kappa_{j}\left(\vec{r},{TE}_{k}\right)$ has been shown to be comparable to the spatially matched filter in the absence of correlated noise.

For multi‐echo data, the combined phase images from each echo time, $\Theta \left(\vec{r},TE_{k}\right)$, can be combined into a single phase image via a weighted sum of unwrapped phase images over *k* echoes. Wu *et al.* proposed the weighting factor for the *k*th echo

(14) Wkr→=TEke−TEkT2*∑k=1KTEke−TEkT2*,

which weights each echo by its SNR 68, as used previously in multi‐echo EPI 69. The weighting factors for *phase difference* methods are given earlier in this section.

### Assessing the quality of combined phase images

In the general absence of a ground truth, assessing the quality of combined phase images is not trivial. In the literature, the absence of open‐ended field lines or the fact that phase images can be unwrapped is often mistakenly seen as being indicative of effective matching and combination of individual channel phase images. However, this takes no account of the loss of SNR that occurs when phase matching is imperfect but has not led to complete destructive loss of signal. We have suggested that the quality of phase matching be represented by the metric *Q* 54.

(15) $$Q=100\times \frac{\left|\sum_{j}M_{j}\;e^{i\left(\varphi_{j,\text{corrected}}\right)}\right|}{\sum_{j}M_{j}}.$$

When the phases of the individual signal vectors are in good agreement (i.e., they are matched), the length of the complex sum of the signals (numerator) is equal to the sum of the length of the individual signal vectors (denominator), i.e. *Q* = 100. *Q* should only be evaluated in voxels containing signal. In background, or noise voxels, the complex sum (the numerator) tends to zero for large *j* (a desirable property), whereas noise is additive in the magnitude sum (the denominator).

### A quantitative comparison of phase combination methods

To allow the phase combination methods described in earlier sections to be compared quantitatively, we applied them to a human *in vivo* multi‐echo data set acquired with a 7 T Siemens MAGNETOM (Siemens Healthcare, Erlangen, Germany) scanner and a 32‐channel Nova Medical coil. This was a triple‐echo gradient‐echo acquisition with a matrix size of 512 × 512 × 208 (voxels of 0.4 × 0.4 × 0.7 mm^3^), monopolar readout and *TE* = [8.0, 14.0, 21.0] ms, *T*_R_ = 26 ms. The bandwidth was 250 Hz/pixel, TA = 10 min 17 s. Phase images were reconstructed according to Equation (13), with the magnitude data as weights, *κ*_*j*_. Noise correlation between coils was not included in the reconstruction. The phase data were combined with no correction for phase offsets (“no correction”, to illustrate the phase combination problem) and with the *phase imaging* methods Roemer, SPM, VRC, MCPC‐3D‐II and COMPOSER and the *phase difference* methods HiP and SVD (see earlier in this section).

The effectiveness of each method was assessed via the quality of phase matching between channels, *Q*, and the contrast‐to‐noise ratio (CNR) between GM and white matter (WM). Exact correspondence of phase values to the (scaled) *B*_0_ fieldmap was not considered essential, as the additional slow phase variations introduced by some methods may be attenuated by later steps in QSM processing 3.

Maps of *Q* were calculated for each echo (for illustration), and the mean of *Q* for each voxel inside the brain over all echoes, *Q*_mean_, for quantification. Histograms of *Q*_mean_ and the median value of *Q*_mean_ over the brain were calculated for each method. GM–WM CNR was also assessed in six ROIs. The GM ROIs were thin strips of cortex; the corresponding WM ROIs were similarly sized and immediately neighboring. The six regions were distributed throughout the brain. Two‐sided paired *t* tests were calculated between the GM–WM CNR measured with the Roemer method (which was treated as the reference) and each of the other approaches.

To assess the effect of phase combination on background‐corrected phase and QSMs, background correction with V‐SHARP was performed with a maximum kernel size of 9 mm and high‐pass‐based regularization of V‐SHARP with a cut‐off frequency of 0.0089 mm^−1^ (parameters determined to be optimal in the review by Özbay *et al.* 70 in this special issue). Phase images were resliced to isotropic resolution before applying V‐SHARP using linear interpolation in the spatial domain. The same internal region for background correction was used for all techniques, defined based on the Roemer‐reconstructed magnitude using FSL BET. Susceptibility maps were reconstructed with a simple *k*‐space‐based algorithm 71.

Phase matching was high throughout the brain with the Roemer method (median *Q*_mean_ of 98.7%), the reference method. The median value of *Q*_mean_ was 18.7% without removal of $\varphi^{0}\left(\vec{r}\right)$ (“No Correction”), with an ostensibly arbitrary distribution of regions of destructive interference between coil signals (visible as low *Q* values (blue) and open‐ended fringeline at arrow). This was improved with SPM (median *Q*_mean_ of 50.0%), where the phase matching was perfect at ROI_M_, at the center of the brain (black square in *Q* map). The directional dependence of the coils on *Q* is apparent, with higher values at the top of the brain, where coils point predominantly in the same direction (head–foot), than at the same distance from ROI_M_ near the bottom of the brain, where they are arranged around the object (see values close to zero in the cerebellum and noise in the phase at arrow), leading to destructive interference. Phase matching was high and uniform with VRC (median *Q*_mean_ of 98.8%). With MCPC‐3D‐II, *Q* values were close to those of VRC (median *Q*_mean_ of 97.8%), although isolated weaknesses from unwrapping errors were apparent at arrow positions. *Q* values were high and uniform with COMPOSER (98.6%) and SVD (99.2%). Within the brain, HiP phase values (not shown) were very similar to those with SVD (typically within 0.1%). *Q* was high in both the image and the background in SVD, as the method maximizes *Q* in every voxel. In summary, phase matching was as effective with VRC, COMPOSER, HiP and SVD as with Roemer (see histograms).

The GM–WM CNR was not significantly different between Roemer and the other *phase imaging* methods: SPM, VRC, MCPC‐3D and COMPOSER. The GM–WM CNR was 41% less on average, however, with the two *phase difference* methods HiP (2.27 ± 0.75) and SVD (2.28 ± 0.75) than with Roemer (3.87 ± 1.06) (a significant difference; *p* = 0.002). The echo times used in this comparison are not ideal for HiP and SVD, however. For the phase difference methods, the first echo time should be as short as possible and the last echo time close to the *T*_2_* of the tissues of interest (see Equation (11)).

**Figure 3:**
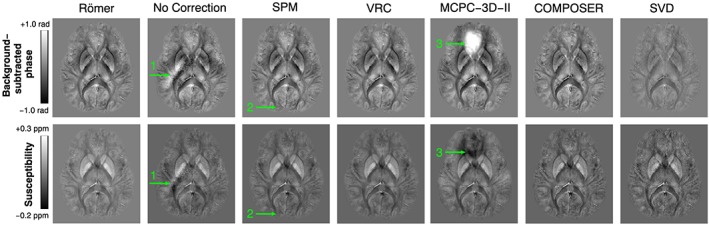
Background‐subtracted phase images and QSMs from the phase combination methods under consideration (Echo 2, average over three slices). Isolated patches of noise (at Arrows 1 and 2) are present in the No Correction and SPM results. In SVD results, phase contrast is reduced and noise increased compared with the Roemer, VRC and COMPOSER methods. MCPC‐3D‐II results are subject to error in frontal areas due to the inclusion of errors in more inferior slices (see arrows in Figure 2) in the V‐SHARP mask.

Background‐corrected phase images and susceptibility maps from Echo 2 of each phase combination method are shown in Figure 3. Isolated patches of noise (at Arrows 1 and 2) are present in the No Correction and, to a lesser extent, SPM results. MCPC‐3D‐II results are subject to errors in frontal areas (see Arrow 3), which arise from inclusion in the internal region of background correction of inferior regions in which there are isolated abrupt phase changes, where unwrapping failed (see arrows in Figure 2). If included in the mask, these cause background field estimation errors. These pervade up to 4 cm superior to the source of the problem, in this case. This effect could be avoided by excluding regions of low *Q* from the mask. SVD results are uniformly noisier than results with the Romer, VRC and COMPOSER methods.

### Practical considerations

The most suitable phase combination method depends on whether a volume reference coil is available, whether multi‐echo measurements are made and whether it is, for a particular application, important to remove all sources of phase other than those arising from susceptibility effects.

The requirements, features and shortcomings of the phase combination approaches described here are summarized in Table 1. In brief, Roemer remains the method of choice if a homogeneous reference coil (e.g. body coil) is available. The Roemer method can also be used if only an inhomogeneous transmit coil can be used to receive signal (e.g. head coil at UHF), although the transmit B1 contributions to the combined phase are not generally harmonic, so are not removed by background correction unless this entails regularization 35, 70.

**Table 1:** Requirements and features of the phase combination methods described in the text ((v)HPF, (very) high‐pass filtered; arb, arbitrary additional phase; inter., intermediate)

	Phase matching (3 T, 7 T)	Computational demand	Phase content	GM–WM contrast/noise	Comments
*Methods requiring a volume reference measurement*					
Roemer/SENSE 8	excellent	low	Δ*B*_0_ + *B*_1_^+^ + B_1_^−^	unmodified	Requires reference scans
*Methods applicable to single‐echo acquisitions*					
Homodyne filt. 45	(fair, poor)	low	vHPF(Δ*B*_0_)	unmodified	Not usable for QSM, fails in regions of high *TE*_*k*_ Δ*B*_0_
Unwrap + filt. 31	(good, fair)	high	HPF(Δ*B*_0_)	unmodified	Requires unwrapping
SPM 48	(fair, poor)	low	Δ*B*_0_ + arb.	reduced in some regions	Matching poor away from center at UHF
VRC 52	excellent	low	Δ*B*_0_ + arb.	unmodified	Residual arbitrary phase, fails where SPM signal =0
MPCP‐3D‐II 54	good	inter.	Δ*B*_0_	unmodified	Requires spatial unwrapping and reference scan
COMPOSER 55	excellent	low	Δ*B*_0_	unmodified	Requires reference scan
*Methods requiring a multi‐echo acquisition*					
Phase diff. 59	excellent	low	Δ*B*_0_	reduced	Reduced CNR
MPCP‐3D‐I 54	good	high	Δ*B*_0_	unmodified	Requires spatial unwrapping
SVD 63	excellent	intermediate	Δ*B*_0_	reduced	Reduced CNR
MAGPI 66	excellent	high	Δ*B*_0_	unmodified	Complex processing, computationally demanding

VRC and COMPOSER proved to be the most effective methods for single‐echo acquisitions where there is no volume reference coil. VRC needs no reference measurement, but (like the SPM method on which it depends) fails where the VRC image yields open‐ended fringelines. COMPOSER is applicable to all coil designs and field strengths but requires a reference scan.

Despite the fact that multi‐echo acquisitions contain the information to identify $\varphi_{j}^{0}\left(\vec{r},TE_{k}\right)$ and $\text{∆}B\left(\vec{r}\right)$ uniquely and without recourse to reference measurements, the methods considered here – HiP, SVD and MAGPI – all have significant shortcomings. HiP and SVD have reduced CNR, particularly if the echo times are not optimized for phase difference calculation, and MAGPI is computationally intensive. We see the need for computationally undemanding solutions to the multi‐echo problem that yield combined images without reducing CNR. The single‐echo methods VRC and COMPOSER can, of course, be applied to multi‐echo data.

## Phase Unwrapping

### Outline of the problem and requirements

Wraps may be present along any of the four dimensions of a multi‐echo data set. The problem of unwrapping is ill posed, as many values of true phase lead to a wrapped value (i.e., it is surjective but non‐injective). In practice, assumptions about spatial or temporal continuity (or both) can be drawn upon to attempt to solve the problem. The success of any method is dependent, however, on how rapidly phase changes between voxels or time points and the level of noise.

Spatial unwrapping methods (see later in this section) draw on the fact that phase generally changes slowly from voxel to voxel unless a wrap has occurred. Spatial approaches can be subdivided into path‐following methods and ‘Laplacian’ unwrapping (see later in this section). Path‐following methods determine a wrap to have occurred if the phase change between two voxels along a path is greater than *π*. The value of +*π* or −*π* is added to all further voxels along the path (depending on whether the phase jump was positive or negative). Unless errors occur, this restores the exact value of the underlying phase. Laplacian unwrapping attempts to identify the unwrapped phase whose local derivatives are most similar to the derivatives of the wrapped phase. This removes discontinuities in the phase but – unless an exact solution is implemented (see later in this section and Appendix A3) – it modifies all phase values in the image in a spatially slowly varying way, introducing some background field suppression 71. This does not propagate into the background‐corrected field 71, so does not affect QSM results, but may prove problematic if the phase is being used quantitatively without background elimination (e.g. to determine $\varphi^{0}\left(\vec{r}\right)$ 55). Finally, phase change between echoes can be used to perform a temporal unwrapping of the data (see later in this section) in a 1D unwrapping approach.

### Spatial phase unwrapping

#### Path‐following methods

An assumption underlying spatial phase unwrapping is that a wrap has occurred between two neighboring voxels if the difference between them is greater than *π*. Algorithms can apply this assumption in a number of ways in one, two or three dimensions, unwrapping the phase along paths guided by the quality of the information that voxels contain 72 and the need to avoid ‘branch cut’ lines 73. Branch cut lines are imposed barriers to paths. They connect problematic path‐dependent ‘residues’, such as occur in 2 × 2 voxels containing the termination of an open‐ended fringe line. The result of unwrapping the phase along any path that does not cross such a branch cut line is independent of the path.

Unwrapping in a higher number of dimensions reduces the sensitivity to noise but increases the complexity of the calculation. Both 2D and 3D algorithms are in use in MRI. The number, complexity and range of these makes it impossible to cover them in depth in this review; the reader is instead referred to Reference 22 for a comprehensive overview. Instead, we try to illustrate the features of some of the unwrapping methods that have become most established in high‐resolution 3D phase imaging, namely PRELUDE 74, Cusack's method 75, BEST‐PATH 76 and PHUN 72. We quantitatively compare the performance of these algorithms with Laplacian and temporal methods in unwrapping highly wrapped and noisy distributions (see later in this section).

#### Laplacian phase unwrapping

Laplacian unwrapping is distinct from other spatial and temporal unwrapping methods because, while it preserves much phase variation in the image, it does not generally yield quantitative phase values. In the light of its quite recent application in MRI (from the field of interferometry), we dedicate additional space to a comprehensive and rigorous explanation of this method to explain its function and features but also explore the potential to extend it to yield quantitative results.

The principal idea behind Laplacian phase unwrapping (as well as its variants) is the observation that, assuming that the unwrapped phase data *φ*^unwrap^ is sufficiently differentiable, some differential operator applied to the unwrapped phase can be obtained from the wrapped phase *φ*^wrap^ 77. As the name suggests, this differential operator is the Laplacian, i.e. $\Delta \varphi =\sum_{i=1}^{n}\frac{{\text{∂}}^{2}\varphi }{\text{∂}x_{i}^{2}}$. Indeed, as exp(i*φ*^unwrap^) =  exp (i*φ*^wrap^), we have, with *ψ* =  exp (i*φ*^wrap^), that

(16) $$\Delta \varphi^{\text{unwrap}}=I\left(\psi^{-1}\Delta \psi \right),$$

where I is the imaginary component. The idea is now to solve the corresponding Poisson equation, i.e. to obtain *φ*^unwrap^ from Δ*φ*^unwrap^. However, at this stage, *φ*^unwrap^ is only determined up to harmonic phase maps, i.e. those whose Laplacian vanishes, so additional information is needed to obtain a solution for the unwrapped phase. One way to incorporate such information is, for instance, to assume that *φ*^unwrap^ is periodic. This may be achieved by mirror extension or, equivalently, by assuming homogeneous Neumann conditions, i.e. $\frac{\text{∂}\varphi^{\text{unwrap}}}{\text{∂}\nu }=0$ on the boundary. Then, *φ*^unwrap^ is uniquely determined up to a global constant, which can, for instance, be determined by fixing *φ*^unwrap^ at a single point or fixing the integral ∫*φ*^unwrap^. Alternatively, some applications such as QSM do not require boundary conditions, as the unwrapped phase only has to be determined up to harmonic components, which will in any case be removed in the subsequent background elimination step (see the review article by Schweser *et al.* in this issue (1) and Reference 70).

On a rectangle or cuboid, one can express the solution of the Poisson equation with the help of Fourier cosine series. Denoting by *F* the Fourier analysis operator and by *F*^−1^ its inverse, the Fourier synthesis operator, we compute, with the help of Equation (16), the Laplacian as

(17) $$\Delta \varphi^{\text{unwrap}}=I\left(\psi^{-1}F^{-1}\left(-{\left|\omega \right|}^{2}F\left(\psi \right)\right)\right),$$

where ${\left|\omega \right|}^{2}=\omega_{1}^{2}+\omega_{2}^{2}+...+\omega_{n}^{2}$ denotes the squared Euclidean norm of the *n*D Fourier indices *ω* = (*ω*_1,_*ω*_2,_ .  . ., *ω*_*n*_). The solution of the Poisson equation may then be computed via

(18) $$\varphi^{\text{unwrap}}=F^{-1}\left(-{\left|\omega \right|}^{-2}F\left(\Delta \varphi^{\text{unwrap}}\right)\right)$$

where, for *ω* = 0, no division should be performed (which fixes ∫*φ*^unwrap^).

While this approach leads to exact phase unwrapping in the continuous setting, its practical application is limited by the fact that we are dealing with discrete data, usually on a uniform grid. Discretization is therefore necessary. In view of Equations (17) and (18), this means replacing *F*and *F*^−1^ by discrete counterparts, as proposed in Reference 77. Doing so either involves the (normalized) discrete cosine transform (DCT) of Type II and its inverse, which is the DCT of Type III 78, i.e., *F* = DCT_II_ and *F*^−1^ = DCT_III_. Alternatively, an equivalent operation such as mirror symmetrization and the fast Fourier transform can be utilized 79. However, Equations (17) and (18) fail in the discrete setting, so applying them only yields an approximation *φ*^apprx^, and, in particular, the difference between *φ*^apprx^ and *φ*^wrap^ will in general not correspond to integer multiples of 2*π*, a property that is satisfied by the exact difference *φ*^unwrap^ − *φ*^wrap^. If one is interested in quantitatively exact results, one has to resort to heuristics. One such approach would be to round the approximation *φ*^apprx^ − *φ*^wrap^ to multiples of 2*π* and add *φ*^wrap^, which gives an approximation to *φ*^unwrap^ that is exact where the rounding is exact. Of course, this may or may not be the case, and the approach works particularly well if the error *φ*^unwrap^ − *φ*^apprx^ was already small. A more sophisticated method is to perform the following congruence operation.

- For *j* = 1 to *N*

Let $h_{j}=\frac{2\mathrm{\pi j}}{N}$.

Let $\varphi_{j}^{\text{apprx}}=\varphi^{\text{apprx}}+h_{j}+∠\left[e^{i\left(\varphi^{\text{wrap}}-\varphi^{\text{apprx}}-h_{j}\right)}\right]$.

Let *D*_*j*_= the number of discontinuities in $\varphi_{j}^{\text{apprx}}$

end.

- Find *j*_min_ for which D is smallest.
- The congruent solution reads $\varphi_{\text{cong}}^{\text{apprx}}=\varphi_{j_{\min}}^{\text{apprx}}$.

There are several possibilities to detect discrete discontinuities; for instance, one can say that a discontinuity occurs if the values in two neighboring pixels differ at least by 2*π*. For noiseless data, the above‐mentioned single‐step congruence operation with *h* = 0 is usually sufficient. However, testing *N* global shifts *h*_1_ ,  …  , *h*_*N*_ may increase the accuracy of the congruence operation in the case of noisy data.

Alternative representations to Equation (16) that are equivalent in the continuous setting give rise to different discrete variants of Equation (17). For instance, in Reference 80, computing Δ*φ*^unwrap^ is proposed according to

(19) Δφunwrap=div−iψ−1∇ψ=F−1iω⋅F∇φwrap,∇φwrap=ψ−1F−1ωFψ,

where *a* ⋅ *b* denotes the scalar product between two vectors. Together with Equation (18), it constitutes another phase‐unwrapping approach of Laplace type. The two variants have in common that they involve only a few deterministic steps with well‐understood mathematical properties. One can show, for instance, that DCT‐based discrete versions of Equations (17), (18), (19) are Lipschitz continuous. This means in particular that these computations are stable with respect to noise, in the sense that noise is amplified up to a finite factor, which turns out to be moderate in practice.

Besides Fourier methods, the approximation of *Δφ*^unwrap^ can also be performed only in the spatial domain by employing finite‐difference techniques. One advantage of these approaches is that the solution step for the discrete Poisson equation can be evaluated in a quantitatively exact manner, i.e., *φ*^unwrap^ may be obtained exactly if one is able to compute its finite‐difference Laplacian Δ_*h*_*φ*^unwrap^. Here, again, one has generally only an approximation Δ_*h*_*φ*^apprx^ available. However, the Poisson inversion process is stable, so the result *φ*^apprx^ is an approximation of *φ*^unwrap^ and the error can be controlled. Furthermore, by employing heuristics, it is possible to compute Δ_*h*_*φ*^unwrap^ for wrapped data with moderate phase wraps, leading to exact results. We refer to Appendix A4 for details.

In practice, applying unweighted finite‐difference techniques (as in Appendix A4) leaves substantial errors, especially in the ventral brain. These are mainly caused by noisy phase data in the background where the magnitude is small. Introducing binary weights *w* (1, tissue; 0, background) and considering the weighted finite‐difference operator div_*h*_(*w*∇_*h*_) instead of the Laplacian (A4, Equation (21)) allow this noisy background phase to be masked out, eliminating the main error source. Such a modification still leads to a linear equation, which can then be represented by a sparse matrix, calling for iterative solution strategies. A particularly well‐suited procedure is the preconditioned conjugate gradient (PCG) method with DCT‐based solution of the unweighted Poisson problem (Equation (23) in Appendix A4) used as preconditioner in each iteration step. The pseudocode for this method is presented in Appendix A3, and a MATLAB implementation is also available 81. We refer the reader to Reference 22 for an extended description. This PCG algorithm is typically characterized by a rapid convergence with well‐defined termination conditions (such as a user‐defined maximum number of iterations and convergence factor). The results obtained with the weighted Laplacian approach by virtue of the PCG method tend to be much closer to the exact solution than those for the unweighted approach. Here, applying the previously mentioned congruence operation also yields further improvements.

In summary, Laplacian phase unwrapping techniques constitute a fast and easy‐to‐implement class of methods that are robust to noise. This robustness generally comes at the cost of obtaining only approximations of the exact phase. The latter issue can, to a certain extent, be resolved by fine‐tuning the discretization, introducing appropriate weighting and fast linear solution techniques as well as employing heuristics and congruence operations. Generally, the methods discussed can be recommended for data sets with moderate to low SNR where it is not crucial to obtain exact quantitative values.

### Temporal phase unwrapping

Temporal unwrapping uses the evolution of the phase over measurements at a number of echo times to remove wraps that are caused by time‐dependent effects, i.e. due to ${\text{∆}B}_{0}\left(\vec{r}\right)$. It therefore requires the information available only in multi‐echo acquisitions, and, until recent extensions, has only been capable of removing wraps that occur over a time greater than the inter‐echo period, and only generates unwrapped phase difference images, not unwrapped phase images.

If no *a priori* information is assumed about the sign of the phase change between two echoes (*φ*_L_ =  − *π* in Equations (4), (5)), then the range of the phase difference that is wrap free is (−*π*, +*π*]. This limit corresponds to a difference between echo times of (2 Δ*f*_MR_)^−1^ s. With a frequency range of interest of ±50 Hz/T in the head, the inter‐echo time needs to be less than 10 ms T, or circa 1.4 ms at 7 T and 3.3 ms at 3 T. This poses an undesirable constraint on the acquisition, and one which may be at or beyond achievable gradient switching rates and physiological d*B*/d*T* limits. This, and the fact that phase difference images have poor noise properties compared with phase images (see the previous section, and Equation (20) in Appendix A2), have seen temporal phase unwrapping largely treated as obsolete for UHF applications. Two approaches have recently been presented that overcome both of these restrictions, however.

In UMPIRE (Unwrapping Multi‐echo Phase Images with Irregular Echo spacings) 82 and MAGPI 83, the authors show that the limitation on the inter‐echo time described above can be overcome by using more than two unequally spaced echoes in a multi‐echo train. In UMPIRE, a short additional delay δ*TE* is inserted between one pair of echoes (e.g. between Echoes 2 and 3). The phase evolution in this period, *Φ*_3 − 2_ − *Φ*_2 − 1_, contains no wraps if δ*TE*, which can be chosen at will, satisfies δTE=12ΔfMR,limit. The phase change *Φ*_3 − 2_ − *Φ*_2 − 1_ is calculated as a first, wrap‐free estimate of Δ*f*_MR_, which is used to identify and remove wraps in *Φ*_3 − 2_ and *Φ*_2 − 1_ and between all further pairs of echoes. Any phase offset is then identified and removed before, in a final step, unwrapping the phase images themselves. In MAGPI, the authors focus on the optimum spacing of echoes to disambiguate the estimation of Δ*f*_MR_, which is identified as the closest L2 distance between the probability distributions representing the possible field map values for each pair of echoes 83.

In general, temporal phase unwrapping has as advantages that it is unaffected – in both time and accuracy – by the complexity of the wrapped topography, and as disadvantages that it is more prone to noise than spatial methods (as it is based on phase differences) and that it is dependent on linear phase evolution, which can be disrupted by tissue characteristics 17, uncompensated flow or idiosyncrasies in the reconstruction.

### An illustrated comparison of phase unwrapping methods

In order to illustrate the features of the spatial, Laplacian and temporal phase unwrapping methods described in the previous section, these were applied to simulated data and *in vivo* multi‐echo phase data acquired at 7 T.

**Figure 4:**
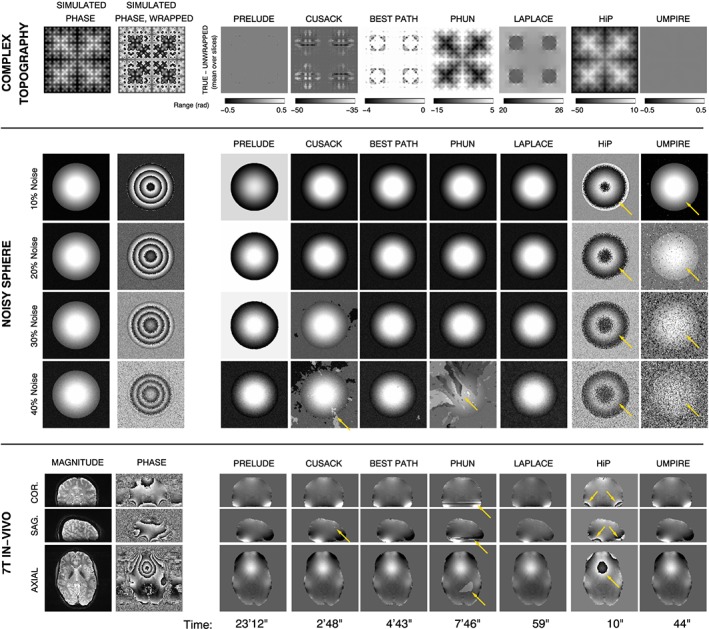
A comparison of the characteristics of the spatial unwrapping methods PRELUDE, CUSACK, BEST PATH and PHUN with Laplacian unwrapping and temporal unwrapping with phase difference imaging (with the HiP) and UMPIRE.

Two types of phase distribution were simulated. The first was a complex phase topography with no noise, the second a simple 3D Gaussian distribution with varying amounts of noise. These were designed to test the methods' ability to resolve complex patterns and to assess unwrapping accuracy in a low‐SNR context respectively. Each phase distribution was modeled as having a purely linear evolution and simulated at three echo times (*TE* = 5, 10, 16 ms – the uneven echo time to allow temporal unwrapping with UMPIRE). More details about the simulated data are given in Reference 82. The complex topographies shown in the upper part of Figure 4 correspond to “Simulated data 2” with complexity level 4 in Reference 82.

The complex topography comprises slices with differing numbers of wraps and complexity. A sample slice is shown at the top left of Figure 4. The mean unwrapping error over all slices is shown for each method. The scale range below each result indicates the size of errors. In the noisy spheres and *in vivo* data, errors are indicated by arrows. The time taken to unwrap all three echoes of the *in vivo* data is reported for each method as the last line of the figure. The 7 T *in vivo* data was acquired with 0.65 mm isotropic voxels, *TE* = [7.4, 14.5, 20.8] ms, that is, with an additional delay of 1 ms between the second and third echoes.

Of the spatial methods, PRELUDE 74 was the most accurate in unwrapping both simulated and *in vivo* data, but was slow where the topographies were noisy. Cusack's method 75 was prone to errors for high noise and suffered isolated errors in *in vivo* data (at arrow position). BEST PATH 76 was relatively robust. PHUN 72 failed to unwrap most slices in the complex shape and some regions of the *in vivo* data and was prone to the most errors at high noise. Unweighted LAPLACE unwrapping 84 was no better than other methods in complex topographies, but was reliable in high‐noise and *in vivo* data. The HiP failed where the limit (2 Δ*f*_MR_)^−1^ was exceeded (at arrows). UMPIRE performed poorly in high noise but had no errors in complex topographies and was free from errors in *in vivo* data above MNI *z* = −14.

**Figure 5:**
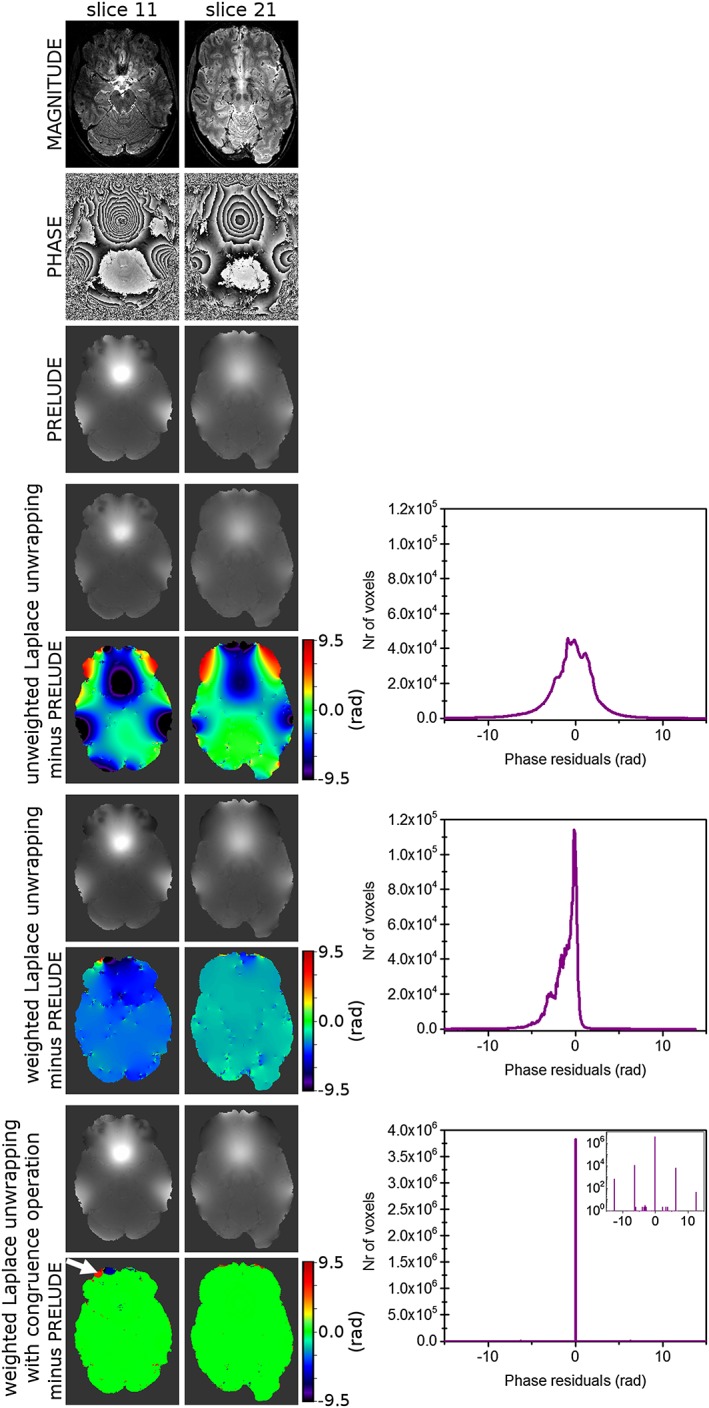
The accuracy of the weighted Laplacian unwrapping compared with an exact PRELUDE solution. The residual phase errors of unweighted Laplacian unwrapping, weighted Laplacian unwrapping (after six iteration steps of PCG) and weighted Laplacian solution with congruence operation are presented in color maps for two inferior slices and histograms over the whole volume.

The amplitude of residual phase ambiguities in the weighted Laplace solution was assessed by comparison with PRELUDE, which, without apparent errors in the slices evaluated, was taken to yield an exact solution. Results for the third echo (with *TE* = 20.8 ms) in two ventral slices are shown in Figure 5. The residual phase errors were assessed for the unweighted Laplace solution, and the weighted Laplace solution without and with the applied congruence operation. Results for two ventral slices are shown in Figure 5. Histograms of phase differences over the whole volume for each of these cases are also presented. Residuals for the unweighted Laplace solution are generally slowly varying. They increase in spatial frequency in the inferior slices, however, where values of ±6 rad are not untypical. Those residues were substantially reduced after six steps of the PCG algorithm for the unweighted Laplace approach, which yields an asymmetric distribution of residual phase errors, with the values mostly within [−5; 1] rad. The congruence algorithm left no residues in most voxels, indicating an exact solution. The exception was a small number of voxels in noisy inferior regions close to the brain boundary, where the residual errors in the weighted Laplace solution were large and led to a wrap in the congruent solution (at the arrow position). Those residuals differ by multiples of 2*π* from the exact solution, illustrated in a logarithmic plot inset in the top right corner of the histogram.

Any assessment of the performance of a phase unwrapping method is dependent on the features of the test object, as well as how the method is implemented and the choice of parameters such as thresholds, seed voxels etc. The reader is referred to References 82, 85 and 86 for other comparative evaluations in a broad range of contexts.

## Conclusion

The phase combination methods compared in this review differ greatly in performance and applicability. Despite a widespread understanding that the Roemer method cannot be used at UHF because of the absence of a body coil, we find that effective phase matching can be achieved with this approach using an inhomogeneous transmit–receive coil. The transmit phase is transferred to the combined image, however, and – as a non‐harmonic phase variation – is not generally removed by background correction. The most effective alternative solutions, applicable to cases in which the transmit element is not engineered to receive signal, were the VRC method and COMPOSER. Multi‐echo approaches were found to match the phase well but be computationally expensive or reduce GM–WM CNR.

A comparison of phase unwrapping methods showed many path‐following spatial methods to be fragile in the presence of rapid phase fluctuations. Temporal unwrapping, while fast, was more prone to errors in low SNR. Laplacian unwrapping was both fast and effective when applied to simulated data with low SNR and *in vivo* data acquired at 7 T.

## Appendix A1 Singular value decomposition

### A

Let *A* be an *n* × *m* complex matrix, *σ* a non‐negative scalar, *u* a non‐zero vector of size *m* and *v* a non‐zero vector of size *n*, such that

Av=σu.

In this case, *u* and *v* are called the left‐singular and right‐singular vectors, respectively, for the singular value *σ*.

The matrix product

$$A=\mathrm{U\Sigma}V^{*}$$

is the SVD of the given matrix *A*.

The diagonal elements of *Σ*, *σ*_*i* , *i*_, are the singular values of *A*, and the columns of *U*, [*u*_*i*_], and *V*, [*v*_*i*_], are the left and right singular vectors, respectively.

The number of non‐zero singular values is the rank *r* of the matrix *A*.

*U* and *V* are unitary matrices (*U*^*^*U* = *U* *U*^*^ = *I*, with *I* the identity matrix) and *Σ* is a diagonal matrix.

For an SVD for an *n* × *m* matrix *A* of rank *r*, the following applies:

the *r* diagonal elements of *Σ* are the square roots of the *r* eigenvalues of *A*^*^*A*

the columns of *V* are the normalized eigenvectors, *v*_*i*_, of *A*^*^*A* to the eigenvalue *σ*_*i*_ and satisfy *A**v*_*i*_ = *σ*_*i*_*u*_*i*_

the columns of *U* are the normalized eigenvectors, *u*_*i*_, of *A**A*^*^ to the eigenvalue *σ*_*i*_ and satisfy *A*^*^*u*_*i*_ = *σ*_*i*_*v*_*i*_.

With these properties, the SVD can be interpreted as a transformation of correlated variables (matrix *A*) into a set of uncorrelated ones (the eigenvectors for different eigenvalues are orthogonal to each other).

## Appendix A2 Derivation of optimum weighting factors in combining phase difference images

### B

The noise of a phase image acquired at an echo time *TE*_*k*_, *φ*_*k*_, is given by

$$\sigma_{\varphi_{k}}=\frac{1}{{\mathrm{SNR}}_{k}}=\frac{e^{{TE}_{k}/{T_{2}}^{*}}}{{\mathrm{SNR}}_{0}}.$$

The noise of the phase difference, *Θ*_*k*1_, between *φ*_*k*_ and *φ*_1_ can be calculated by error propagation:

σΘk1=∂Θk1∂φk2σφk2+∂Θk1∂φ12σφ12=eTEk/T2*SNR01+e2TE1−TEk/T2*.

The noise of a frequency map, $\omega_{k1}=\Theta_{k1}/{\mathrm{TE}}_{k}-{\mathrm{TE}}_{1}$ can then be expressed by

(20) σωk1=1SNR0TEk−TE1e2TEk/T2*+e2TE1/T2*.

Alternatively, because *T*_2_* is unknown, although it is known that the image SNR is not given by image amplitude (because the noise is spatially varying in the presence of parallel imaging), the standard deviation of the noise on the fieldmap across echo times will have the following proportionality:

$$\sigma_{\omega_{k1}}\propto \frac{1}{\left({TE}_{k}-{TE}_{1}\right)}\sqrt{\frac{1}{M_{k}^{2}}+\frac{1}{M_{1}^{2}}}=\frac{1}{\left({TE}_{k}-{TE}_{1}\right)}\sqrt{\frac{M_{1}^{2}+M_{k}^{2}}{M_{k}^{2}M_{1}^{2}}}.$$

To minimize the error in a least square sense, a weighted average with the inverse of the variance, 1σ2, has to be made:

$$〈\omega 〉=\frac{\sum_{k=2}^{K}\frac{1}{{\sigma_{\omega_{k1}}}^{2}}\omega_{k1}}{\sum_{k=2}^{K}\frac{1}{{\sigma_{\omega_{k1}}}^{2}}}=\frac{\sum_{k=2}^{K}\frac{{\left({TE}_{k}-{TE}_{1}\right)}^{2}M_{k}^{2}M_{1}^{2}}{M_{1}^{2}+M_{k}^{2}}\omega_{k1}}{\sum_{k=2}^{K}\frac{{\left({TE}_{k}-{TE}_{1}\right)}^{2}M_{k}^{2}M_{1}^{2}}{M_{1}^{2}+M_{k}^{2}}}$$

$$=\frac{\sum_{k=2}^{K}\frac{{\left({\mathrm{TE}}_{k}-{TE}_{1}\right)}^{2}M_{k}^{2}}{M_{1}^{2}+M_{k}^{2}}\omega_{k1}}{\sum_{k=2}^{K}\frac{{\left({\mathrm{TE}}_{k}-{TE}_{1}\right)}^{2}M_{k}^{2}}{M_{1}^{2}+M_{k}^{2}}}=\frac{\sum_{k=2}^{K}\frac{\left({\mathrm{TE}}_{k}-{TE}_{1}\right)M_{k}^{2}}{M_{1}^{2}+M_{k}^{2}}\Theta_{k1}\;}{\sum_{k=2}^{K}\frac{{\left({TE}_{k}-{TE}_{1}\right)}^{2}M_{k}^{2}}{M_{1}^{2}+M_{k}^{2}}},$$

the result in Equation (11).

## Appendix A3 Preconditioned conjugate gradient algorithm for weighted Laplacian unwrapping

### C

The main task for weighted Laplacian unwrapping is to solve the finite‐difference equation *L*_*h*_*φ*^*apprx*^ = *b*^*wrap*^, where the forward operator is given by *L*_*h*_ =  − *div*_*h*_(*w*∇_*h*_) with binary weight *w*, *b*^*wrap*^ =  − *div*_*h*_(*w*∇_*h*_*φ*^*apprx*^) and ∇_*h*_*φ*^*apprx*^ is given by Equation (24). Here, the operator *L*_*h*_ can be represented by a sparse matrix. The PCG solution to this problem can be described as a pseudocode in the following steps.

Initialize the iteration step: *j* = 1, the convergence factor: *ϵ*_*j* = 1_ = 1, and the solution matrix: $\varphi_{1}^{\text{apprx}}=0$.

Compute the weighted Laplacian of the wrapped phase *b*^*wrap*^ (as described above), set *r*_1_ = *b*^*wrap*^.

Do while *j* < (maxno of iterations) and *ϵ*_*j*_ < *ϵ*_*converged*_

Obtain preconditioner, i.e. solve ${\text{∆}}_{h}\varphi_{j}^{\text{precon}}=-r_{j}$ with the help of Equation (23) in Appendix A4, where $I\left(\psi^{-1}\Delta_{h}\psi \right)$ is substituted with −*r*_*j*_.

Let $t_{j}=\sum_{k,l}{\left(r_{j}\right)}_{k,l}{\left(\varphi_{j}^{\text{precon}}\right)}_{k,l}$, where *k* , *l* represent indices in 2D space.

If *j* = 1, then

$$p_{j}=\varphi_{j}^{\text{precon}}$$

else

$\beta_{j}=\frac{t_{j}}{t_{j-1}}$ ,

$p_{j}=\varphi_{j}^{\text{precon}}+\beta_{j}\;p_{j-1}$

end.

Calculate the weighted Laplacian of *p*_*j*_, i.e. *q*_*j*_ = *L*_*h*_*p*_*j*_.

Let $\alpha_{j}=\frac{t_{j}}{\sum_{k.l}{\left(p_{j}\right)}_{k,l}\cdot {\left(q_{j}\right)}_{k,l}}$.

Let the solution at step *j* be: $\varphi_{j+1}^{\text{apprx}}=\varphi_{j}^{\text{apprx}}+\alpha_{j}\;p_{j}$.

Let *r*_*j* + 1_ = *r*_*j*_ − *α*_*j*_ *q*_*j*_.

Let $\epsilon_{j+1}=\frac{\left\|r_{j+1}\right\|}{\left\|r_{j}\right\|}$ .

Replace *j* with *j* + 1.

end.

## Appendix A4 Finite‐difference techniques for Laplacian phase unwrapping

### D

Besides Fourier methods, the approximation of Δ*φ*^unwrap^ can also be performed only in the spatial domain. Here, the most common way of approximating the Laplacian is by finite‐difference stencils, which read, for the 2D case and resolution *h*_*x*_ > 0 and *h*_*y*_ > 0, as

(21) $${\left(\Delta_{h}\varphi \right)}_{k,l}=\frac{1}{h_{x}^{2}}\left(\varphi_{k-1,l}-2\varphi_{k,l}+\varphi_{k+1,l}\right)+\frac{1}{h_{y}^{2}}\left(\varphi_{k,l-1}-2\varphi_{k,l}+\varphi_{k,l+1}\right)$$

with a straightforward adaptation for higher dimensions 86. Substitution of this into Equation (16) leads to the discrete Poisson problem for the approximate unwrapped phase *φ*^apprx^ according to

(22) $$\Delta_{h}\varphi^{\text{apprx}}=ℑ\left(\psi^{-1}\Delta_{h}\psi \right),\;\frac{\partial \varphi^{\text{apprx}}}{\partial \nu }=0$$

where the boundary condition also has to be understood in a discrete finite‐difference sense. Again, on rectangular domains, Equation (22) is solvable with transform techniques that diagonalize the discrete Laplace operator. It turns out that that can again be achieved with the DCT of Type II, because of the homogeneous Neumann conditions. This leads to the solution formula

(23) $$\begin{array}{c} \overset{⌢}{\Delta_{h}\varphi }\text{apprx}={\mathrm{DCT}}_{\mathrm{II}}\left(ℑ\left(\psi^{-1}\Delta_{h}\psi \right)\right),\\ \varphi^{\text{apprx}}={\mathrm{DCT}}_{\mathrm{III}}\left(\frac{{\overset{⌢}{\Delta_{h}\varphi }}^{\text{apprx}}}{4\sin^{2}\left(\omega_{1}\pi /\left(2N\right)\right)+4\sin^{2}\left(\omega_{2}\pi /\left(2M\right)\right)}\right), \end{array}$$

where (*N*, *M*) denotes the size of the data and, as before, no division should be performed for *ω* = 0. It is worth noting that here, in contrast to the discrete version of Equation (18), the solution step for the discrete Poisson equation is numerically exact 87 and the only source of error is the fact that in general *Δ*_*h*_*φ*^unwrap^ ≠ *Δ*_*h*_*φ*^apprx^. Nonetheless, the solution operator transforms local errors into global errors, meaning that, even in the case where the above inequality holds in only one pixel, we have *φ*^unwrap^ ≠ *φ*^apprx^ in every pixel; that is, the solution will in general be exact nowhere, as is also the case for classical Fourier approaches. Nevertheless, one can say that, when the discrete version of *φ*^unwrap^ originates from a continuously defined smooth function, the error to the exact solution can be estimated according to $\left|{\left(\Delta_{h}\varphi^{\text{unwrap}}\right)}_{k,l}-{\left(\Delta_{h}\varphi^{\text{apprx}}\right)}_{k,l}\right|⩽C\left(h_{x}^{2}+h_{y}^{2}\right)$, where *C* > 0 is a constant within a reasonable range. As solving the discrete Poisson equation is stable with respect to the resolution 88, it also holds that $\left|\varphi_{k,l}^{\text{unwrap}}-\varphi_{k,l}^{\text{apprx}}\right|⩽C\prime \left(h_{x}^{2}+h_{y}^{2}\right)$ for some *C* ′  > 0 not depending on *h*_*x*_and *h*_*y*_, i.e., the pixelwise error can be controlled and vanishes with a quadratic rate in the resolution. The latter is, however, only of theoretical interest since in practice phase data cannot be measured with arbitrary resolution.

Furthermore, the spatial approximation of *Δ*_*h*_*φ*^unwrap^ makes it possible to obtain quantitatively exact results in some cases using Laplacian methods. The general strategy here is to replace the computation of *Δ*_*h*_*φ*^apprx^ in Equation (22) with the aim that it matches or at least gives a better approximation to the unknown *Δ*_*h*_*φ*^unwrap^. An idea in this direction is founded on the observation that, in the continuous setting, one can already obtain the gradient of *φ*^unwrap^ from *φ*^wrap^ via ∇*φ*^unwrap^ =  − i*ψ*^−1^∇*ψ* (see also Equation (19)). As before, this does not transfer to discrete data, however, suggesting computation of an approximation of the discrete exact gradient ∇_*h*_*φ*^unwrap^ from *φ*^wrap^. Indeed, assuming the lowest possible phase change between two pixels, for instance, one arrives at

(24) $${\left(\nabla_{h}\varphi^{\text{apprx}}\right)}_{k,l}=\left(\begin{array}{c} h_{x}^{-1}\arg\left(\psi_{k+1,l}/\psi_{k,l}\right)\\ h_{y}^{-1}\arg\left(\psi_{k,l+1}/\psi_{k,l}\right) \end{array}\right)$$

where arg denotes the argument of a complex number, i.e., the angle that lies here in the interval [ − *π* , *π*). If the phase change in *φ*^unwrap^ is indeed in this interval, the discrete gradient will be exact. Consequently, applying the discrete divergence gives the discrete Laplacian, i.e. *Δ*_*h*_*φ*^apprx^ = div_*h*_(∇_*h*_*φ*^apprx^), which would also be exact as well as the resulting *φ*^apprx^, as solving the discrete Poisson problem does not introduce errors. However, in turn, any deviation of ∇_*h*_*φ*^apprx^ according to Equation (22) from ∇_*h*_*φ*^unwrap^ is an integer multiple of 2*π*/*h*_*x*_ or 2*π*/*h*_*y*_ and causes, as before, a global deviation of *φ*^apprx^ from *φ*^unwrap^, again even in the case where this occurs only in a single pixel.

As for the Fourier‐type methods, the spatial variants consist of only few computational steps. Lipschitz continuity can again be ensured for Equation (23), but, in contrast, fails for Equation (24) due to the fact the complex argument is not continuous. Nevertheless, as argued above, employing a suitably adapted version of Equation (23) leads to exact results if ∇_*h*_*φ*^apprx^ is exact, and this might still be the case in the presence of noise.
